# Suicidal Ideation and Suicide Attempts in Anxious or Depressed Family Caregivers of Patients with Cancer: A Nationwide Survey in Korea

**DOI:** 10.1371/journal.pone.0060230

**Published:** 2013-04-02

**Authors:** Boyoung Park, So Young Kim, Ji-Yeon Shin, Robert W. Sanson-Fisher, Dong Wook Shin, Juhee Cho, Jong Hyock Park

**Affiliations:** 1 National Cancer Control Institute, National Cancer Center, Goyang, Korea; 2 Priority Research Centre for Health Behaviour, University of Newcastle & Hunter Medical Research Institute, New South Wales, Australia; 3 Seoul National University Hospital and Seoul National University Cancer Hospital, Seoul, Korea; 4 Sungkyunkwan University School of Medicine, Seoul, Korea; University of Rochester, United States of America

## Abstract

**Purpose:**

To describe the prevalence of suicidal ideation and suicide attempts in family caregivers (FCs) of patients with cancer and to identify the factors associated with suicidal ideation and suicide attempts in FCs with anxiety or depression.

**Methods:**

A national, multicenter survey administered to 897 FCs asked questions concerning suicidal ideation and suicide attempts during the previous year and assessed anxiety, depression, socio–demographic factors, caregiving burden, patient factors, and quality of life (QOL).

**Results:**

A total of 17.7% FCs reported suicidal ideation, and 2.8% had attempted suicide during the previous year. Among FCs with anxiety, 31.9% had suicidal ideation and 4.7% attempted suicide; the corresponding values for FCs with depression were 20.4% and 3.3%, respectively. Compared with FCs without anxiety and depression, FCs with anxiety or depression showed a higher adjusted odds ratios (aOR) for suicidal ideation (aOR  = 4.07 and 1.93, respectively) and attempts (OR  = 3.00 and 2.43, respectively). Among FCs with anxiety or depression, being female, unmarried, unemployed during caregiving, and having a low QOL were associated with increased odds of suicidal ideation. FCs with anxiety who became unemployed during caregiving constituted a high-risk group for suicide. Being unmarried and having a low QOL with respect to financial matters were associated with increased suicide attempts among FCs with depression.

**Conclusion:**

FCs with anxiety or depression were at high risk of suicide. Interventions to enhance social support and to improve perceived QOL may help prevent suicide and manage suicidal ideation in FCs with anxiety or depression.

## Introduction

Cancer is a significant cause of physical, emotional, and practical problems for family caregivers (FCs) of patients with cancer as well as for the patients themselves. The primary care setting for cancer patients has shifted from the hospital to the home as a result of decreased hospital stays, increased outpatient treatment, longer survival, and patients’ desire to be cared for at home. As a result, the burden on informal caregivers, who are primarily FCs, has increased. In the face of these increasing challenges and responsibilities, FCs often experience a decline in physical health, personal welfare, and well-being as well as psychological distress and a decline in mental health. [1], [2].

The most common psychological problems experienced by FCs are anxiety and depression, and many have reported contemplating suicide. [3] Several previous studies of the mental health of patients with cancer have reported that the risk of suicide in this group is higher than that in the general population and that vulnerability factors for suicide, such as depression, isolation, fear of being a burden, and poor prognosis, [4]–[6] are common in patients with cancer. However, despite increasing interest in the psychological stress suffered by caregivers, few studies have investigated the risk of suicide in FCs of patients with cancer, who are often the primary caregivers and who are subjected to an increasing burden of responsibility. [7] Depression and anxiety, which are more common and severe in the FCs of patients with cancer than in cancer patients themselves, [8] are significant psychological problems in their own right and are associated with increased suicidal ideation. [9], [10].

The present study aimed to investigate the prevalence of suicidal ideation and suicide attempts among FCs of patients with cancer and to identify factors associated with suicidal ideation and suicide attempts in FCs representing a nationally representative study population. To our knowledge, the present study is the first reported investigation of issues related to suicide in the FCs of patients with cancer.

## Materials and Methods

### Study Design and Population

A multicenter nationwide survey was conducted between July and September 2011 as part of a government program to develop comprehensive supportive care in the Republic of Korea. The program was implemented and funded by the Korean Ministry of Health and Welfare and the Korean National Cancer Center (NCC). The NCC and nine regional cancer centers designated by the Korean government across all provinces of Korea participated. In each center, 100 patients older than 18 years of age who had been diagnosed with cancer more than 4 months prior to the study were enrolled along with their caregivers. Among 1,309 patient–caregiver dyads invited, 990 agreed to participate and completed the survey according to the instructions (75.6% participation rate). All participants were thoroughly informed about the content and aims of the study by trained research coordinators, and all participating patients and caregivers signed consent forms. The survey questionnaires were self-administered, and most patients and caregivers completed them without help. Among 990 patient–caregiver dyads, 20 with missing variables for either anxiety or depression and 73 in which the caregivers were not family members of the patient were excluded from the study, leaving 897 patient–FC dyads in the final analysis. This study was approved by the Institutional Review Board of the National Cancer Center, Korea.

### Measures

The FCs were asked about suicidal ideation and suicide attempts during the previous year. Anxiety and depression were assessed using the Hospital Anxiety and Depression Scale (HADS), [11] which has been extensively validated in the Korean population. [12] The HADS consists of two subscales to distinguish between anxiety and depression. Participants whose subscale scores were 8 or higher were placed in the anxiety or depression group, as appropriate.

FCs provided information on demographic characteristics such as sex, age, marital status, education, employment status during caregiving, monthly household income, comorbidity, and relationship to the patient. Monthly household incomes of <2000 US dollars were included in the lower 30% of monthly household incomes in Korea, incomes of 2000–2999 US dollars were included in the middle 30–60%, and incomes of ≥3000 US dollars were included in the upper 40%. The comorbidity of FCs was assessed by asking them if they suffered from any chronic diseases. Furthermore, we collected information concerning caregiving burden, including the number of years spent caregiving since diagnosis and the number of hours spent caregiving per day. FCs’ quality of life (QOL) was measured using the Korean version of the Caregiver Quality of Life Index-Cancer (CQOLC-K) scale. [13] Patients provided information about demographic factors such as sex, age, and education. Additionally, medical information, including the Surveillance, Epidemiology, and End Results (SEER) stage, comorbid conditions, time since diagnosis, and care setting were retrieved for each patient from the hospital information system of each participating center. The QOL of patients was measured using the Korean version of the European Organization for Research and Treatment of Cancer Quality of Life Core Questionnaire (EORTC-QLQ-C30) [14] EORTC-QLQ-C30 global QOL scale was used.

### Statistical Analysis

The distributions of FC and patient characteristics are presented as numbers and percentages. The prevalence of suicidal ideation and suicide attempts during the previous year among FCs with or without anxiety or depression were calculated as proportions, and then odds ratios (ORs) were calculated after adjusting for variables related to demographic characteristics, caregiving burden, and QOL of FCs, and patient characteristics.

Factors associated with suicidal ideation and suicide attempts in the last year among FCs with anxiety or depression were identified by calculating ORs and 95% confidence intervals (CI) using univariate logistic regression analysis. To avoid over-adjustment by including an excessive number of variables, [15] variables with a *p*-value <0.2 were included in the multivariate logistic regression analyses using a forward variable selection method with an entry level of *p*  = 0.1. All statistical analyses were performed using SAS statistical software (version 9.1; SAS Institute Inc., Cary, North Carolina, USA).

## Results

Table 1 presents data on the characteristics of FCs and patients, the caregiving burden, and the QOL of FCs according to CQOLC-K variables. About 60% of FCs were female, and 58.9% were spouses of cancer patients. The FCs who were not spouses of cancer patients were patients’ children (20.3%), parents (16.2%), and siblings (4.6%). Among the 897 FCs, 342 (38.1%) screened positive for anxiety, and depression was present in 737 (82.2%).

**Table 1:** Demographic characteristics of patients and family caregivers.

Characteristics	Caregivers (*n* = 897)	
	N	%
Caregiver		
Sex		
Male	356	39.7
Female	541	60.3
Age, years		
<50	387	43.1
≥50	510	56.9
Marital status		
Married	715	79.7
Other	182	20.3
Education		
<High school	233	26.1
High school	318	35.6
≥College	342	38.3
Employment status during caregiving		
Unemployed → Unemployed	399	46.3
Employed → Employed	298	34.6
Employed → Unemployed	165	19.1
Monthly household income		
<2000 US dollars	434	48.5
2000–2999 US dollars	296	33.1
≥3000 US dollars	164	18.3
Comorbidity		
No	654	75.4
Yes	213	24.6
Relationship to patient		
Spouse	528	58.9
Other	369	41.1
Patient		
Sex		
Male	419	46.7
Female	478	53.3
Age, years		
<50	201	22.4
≥50	696	77.6
Education		
<High school	399	44.6
High school	276	30.9
≥College	219	24.5
SEER stage		
In situ, local	298	34.6
Regional, distant	564	65.4
Comorbidity		
No	562	62.6
Yes	335	37.4
Time since diagnosis		
<1 year	531	59.2
≥1 year	366	40.8
Care setting		
Inpatient	224	25.0
Outpatient	673	75.0
Global quality of life (EORTC QLQ-C30)		
Good	435	48.5
Poor	462	51.5
Caregiving burden		
Duration of caregiving		
<1 year	401	45.4
1 year	233	26.4
≥2 years	250	28.3
Number of hours per day spent caregiving		
≤1	339	37.9
2–5	182	20.3
≥6	374	41.8
Quality of life of FCs (CQOLC-K)		
Burden		
Mean (SD)	17.3(8.5)	
Good	431	48.7
Poor	455	51.3
Disturbance		
Mean (SD)	9.3(5.8)	
Good	401	45.2
Poor	487	54.8
Positive adaptation		
Mean (SD)	14.5(5.2)	
Good	473	53.2
Poor	417	46.8
Financial concern		
Mean (SD)	4.7(3.5)	
Good	446	50.0
Poor	446	50.0

Abbreviations: *SD*, standard deviation; EORTC QLQ-C30, European Organization for Research and Treatment of Cancer Quality of Life Core Questionnaire; CQOLC-K, Korean version of the Caregiver Quality of Life Index-Cancer.

**Figure 1:**
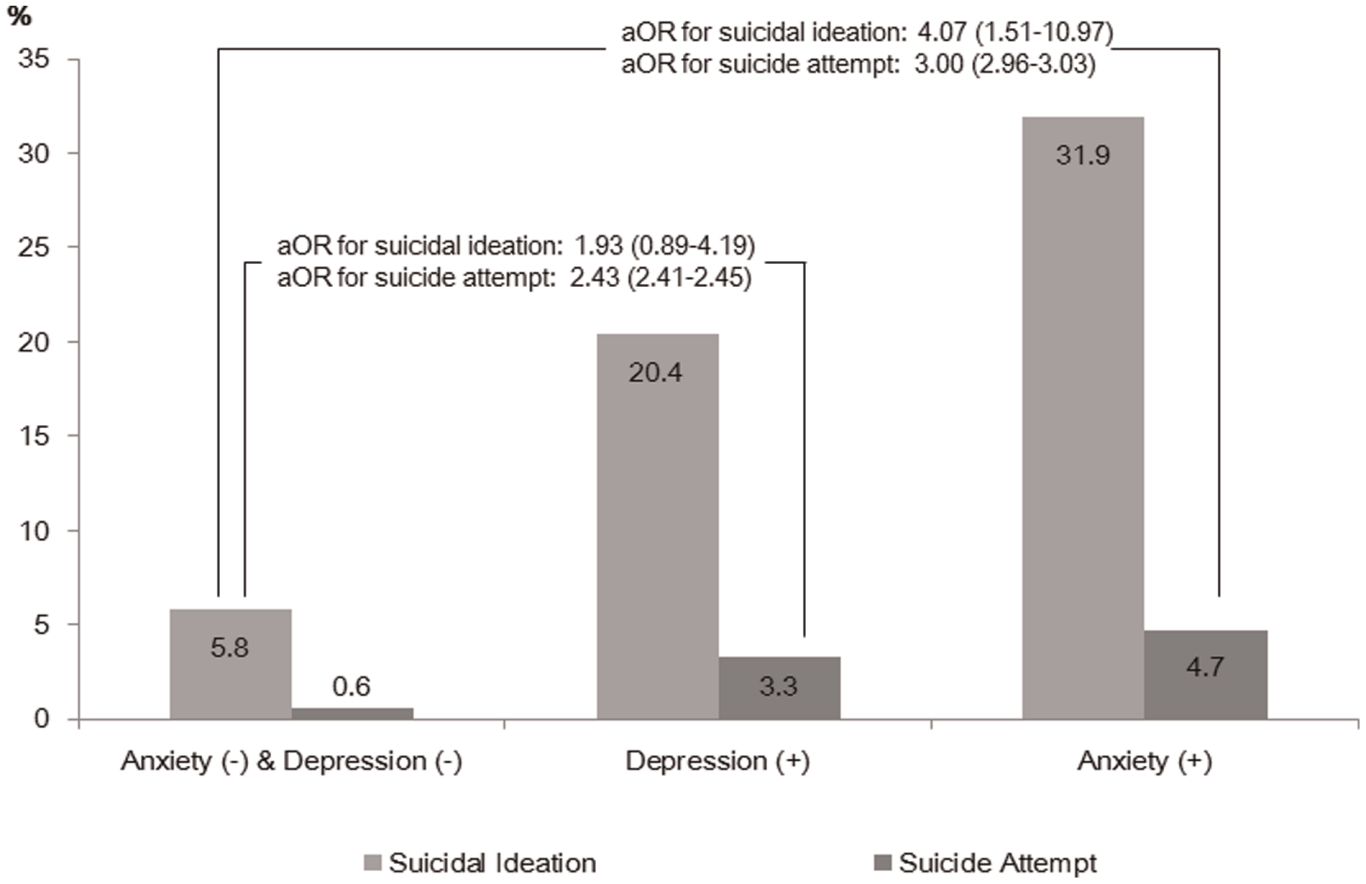
Prevalence and odds ratios of suicidal ideation and suicide attempts in family caregivers with or without anxiety and depression. Abbreviation: aOR, adjusted odds ratio for demographic characteristics of family caregivers, caregiving burden, quality of life of family caregivers, and patients’ characteristics.

We found 17.7% (*n*  = 159) of FCs reported suicidal ideation, and 2.8% (*n*  = 25) had attempted suicide during the previous year. Among FCs with anxiety, 31.9% (*n*  = 109) reported suicidal ideation, and 4.7% (*n*  = 16) had attempted suicide, which was significantly higher than the percentage of FCs without anxiety and depression (suicidal ideation, 5.8%, adjusted OR  = 4.07 (95% CI, 1.51 to 10.97); attempted suicide, 0.6%, adjusted OR  = 3.00, (95% CI, 2.96 to 3.03); Fig. 1). Moreover, 20.4% (*n*  = 150) of FCs with depression experienced suicidal ideation, and 3.3% (*n*  = 24) had attempted suicide. These values were higher than those for FCs without anxiety and depression (suicide ideation, adjusted OR  = 1.93 (95% CI, 0.89 to 4.19); suicide attempt, adjusted OR  = 2.43 (95% CI, 2.41 to 2.45); Fig. 1); however, the association between suicidal ideation and depression was not significant.

Variables associated with suicidal ideation and suicide attempts with *p*-values <0.20 in the univariate analysis (data not shown) were included in the multivariate analysis using the forward selection method. Among FCs with anxiety, being female, being unmarried, being unemployed during caregiving, and having a low QOL on the CQOLC-K positive-adaptation variable were associated with an increased risk of suicidal ideation. The adjusted ORs were as follows (Table 2): being female, 1.96 (95% CI, 1.06 to 3.63); being unmarried, 2.26 (95% CI, 1.19 to 4.31); being unemployed during caregiving, 1.97 (95% CI, 1.09 to 3.55); and having a poor QOL on the CQOLC-K positive-adaptation variable, 1.67 (95% CI, 1.01 to 1.76).

**Table 2:** Multivariate analysis of factors associated with suicidal ideation in family caregivers with anxiety or depression.

Characteristics		Anxiety (*n* = 342)				Depression (*n* = 737)		
	OR			95% CI	OR		95% CI	
Caregiver								
Sex								
Male	1				1			
Female	1.96			1.06 to 3.63	2.08		1.28 to 3.38	
Marital status								
Married	1				1			
Other	2.26			1.19 to 4.31	1.97		1.22 to 3.19	
Employment status during caregiving								
Unemployed→Unemployed	1				1			
Employed→Employed	0.59			0.29 to 1.21	0.56		0.32 to 0.99	
Employed→Unemployed	1.97			1.09 to 3.55	1.76		1.08 to 2.87	
Patient								
Age, years								
<50					1			
≥50					0.60		0.38 to 0.96	
Quality of life of family caregivers (CQOLC-K)								
Burden								
Good					1			
Poor					2.29		1.38 to 3.82	
Disturbance								
Good					1			
Poor					2.13		1.26 to 3.60	
Positive adaptation								
Good			1		1			
Poor			1.67	1.01 to 2.76	2.05		1.34 to 3.13	

Abbreviations: OR, odds ratio; CI, confidence interval; CQOLC-K, Korean version of the Caregiver Quality of Life Index-Cancer.

* A higher score represents a higher quality of life related to caregiving (poor ≤ mean; good>mean).

Variables associated with increased ORs of suicidal ideation among FCs with depression were being female (OR  = 2.08 (95% CI, 1.28 to 3.38)), being unmarried, (OR  = 1.97 (95% CI, 1.22 to 3.19)), being unemployed during caregiving (OR  = 1.76 (95% CI, 1.08 to 2.87)), and having a poor QOL according to the CQOLC-K burden variables (OR  = 2.29 (95% CI, 1.38 to 3.82)), disturbance (OR  = 2.13 (95% CI, 1.26 to 3.60)), and positive adaptation (OR  = 2.05 (95% CI, 1.34 to 3.13). Being employed during caregiving and caring for an older patient were associated with decreased suicidal ideation (OR  = 0.56 (95% CI, 0.32 to 0.99) and OR  = 0.60 (95% CI, 0.38 to 0.96), respectively, Table 2).

Being unemployed during caregiving increased suicide attempts in FCs with anxiety (OR  = 3.27, (95% CI, 1.12 to 9.56)), whereas being unmarried and having a low QOL related to financial matters according to the CQOLC-K were associated with depression in FCs (OR  = 3.59 (95% CI 1.54 to 8.35) and OR  = 2.73 (95% CI, 1.06 to 7.02), respectively, Table 3).

**Table 3:** Multivariate analysis of factors associated with suicide attempts in family caregivers with anxiety or depression.

Characteristics	Anxiety		Depression	
	OR	95% CI	OR	95% CI
Caregiver				
Marital status				
Married			1	
Other			3.59	1.54 to 8.35
Employment status during caregiving				
Unemployed→Unemployed	1			
Employed → Employed	0.30	0.04 to 2.57		
Employed → Unemployed	3.27	1.12 to 9.56		
Quality of life of family caregivers (CQOLC-K)				
Financial concern				
Good			1	
Poor			2.73	1.06 to 7.02

Abbreviations: OR, odds ratio; CI, confidence interval; CQOLC-K, Korean version of the Caregiver Quality of Life Index-Cancer.

* A higher score represents a higher quality of life related to caregiving (poor ≤ mean; good>mean).

## Discussion

The present nationwide study examined the prevalence of suicidal ideation and suicide attempts in FCs of patients with cancer. Moreover, we identified FCs at high risk for suicide by identifying the factors associated with suicidal ideation and suicide attempts in FCs. We found that FCs with anxiety or depression had higher rates of suicidal ideation and suicide attempts than did FCs without anxiety and depression. Being female, unmarried, unemployed during caregiving, or having a low QOL increased suicidal ideation among FCs who experienced anxiety or depression. Conversely, being employed during caregiving and caring for an older patient were associated with decreased suicidal ideation among FCs with depression. Being unemployed during caregiving constituted a high-risk factor for suicide attempts among FCs with anxiety, and being unmarried and having a low QOL was associated with increased suicide attempts among FCs with depression.

Depression and anxiety symptoms are significant and independent risk factors for suicidal ideation and suicide attempts, [9], [10], [16] and suicidal ideation is the strongest predictor of suicide attempts. [17] Our results showed that the 1-year prevalence of suicidal ideation and suicide attempts in FCs was 17.7% and 2.8%, respectively–higher than that in the general population, medical inpatients, [18], [19] and patients with cancer. [20] However, when we grouped FCs according to their anxiety or depression status, we found that the rate of suicidal ideation and suicide attempts in FCs without anxiety and depression was similar to that in the general population in Korea (5.8% *vs.* 3.7% for suicidal ideation and 0.6% *vs.* 0.7% for suicide attempts, respectively). [18] We confirmed that anxiety and depression were independently and significantly associated with suicide in FCs. This finding is consistent with those of previous studies in patients with cancer. [4], [21] Furthermore, the magnitude of the ORs for anxiety and depression for suicidal ideation and suicide attempts were higher in FCs than in the general population. [9], [10], [22] Among FCs who experienced suicidal ideation, 11.5% had attempted suicide during the previous year (data not shown). Thus, FCs with anxiety or depression should be closely monitored for suicidal intentions.

An important finding of the present study is that unemployment during caregiving was associated with suicidal ideation and suicide attempts in FCs with anxiety and associated with suicidal ideation in FCs with depression. Previous studies of the general population have consistently shown that change in employment or loss of employment is associated with an increased risk of suicide. [23]–[25] Unemployment negatively affected the caregiving burden of FCs by decreasing income and increasing the financial burden, as has been observed in the general population. [26] Moreover, being unemployed may be related to a negative self-image. [26] Combining work and caregiving may have varying effects on FCs. The “scarcity hypothesis” asserts that multiple roles can deplete caregivers’ energy and resources, resulting in adverse outcomes. [27], [28] The “expansion hypothesis” argues that multiple roles offer opportunities for prestige, recognition, and financial reward that could increase caregivers’ well-being. Full-time caregivers may have limited social contact and lose the social roles they held outside of caregiving. [28], The positive aspects of combining work and caregiving, such as a sense of accomplishment, enhanced interpersonal relationships, and opportunities to compensate for limitations experienced in each separate role, have been reported to outweigh the negative aspects. [30] Our results indicate that maintaining employment during caregiving is particularly important for the psychological well-being of FCs with anxiety or depression as this group is at high risk for suicidal ideation and suicide attempts.

Our finding that being female, being unmarried, and having a low QOL were associated with suicidal ideation and suicide attempts is consistent with those of previous studies of the general population or patients with cancer. [4], [9], [16], [31] Previous studies have reported that females had a higher rate of suicidal ideation [4], [16] and exhibited more suicidal behaviors than did males. [32] Being unmarried has been associated with less social support, resulting in greater frustration, more suicidal ideation, and more suicide attempts. [31] A low QOL is associated with low individual satisfaction and decreased capacity to cope with stress. [9] However, we did not find an association between suicidal ideation or suicide attempts and socio-demographic factors such as education, income, or comorbidity, which earlier studies identified as significant risk factors. [9], [33] Furthermore, the medical condition of the patient, which is associated with suicide in patients with cancer, was not associated with suicidal ideation or suicide attempts in FCs. [4], [6], [20].

The present study has several limitations. First, only FCs who visited the centers with patients were assessed. Second, the cross-sectional design used in the present study does not allow us to attribute causality to the findings. Third, the 10 cancer centers (one Korean National Cancer Center and nine regional cancer centers) are national hospitals, and private hospitals were not included. Although patients treated at national hospitals may differ from those treated at private hospitals, we were unable to measure these differences. Additionally, we did not assess differences existed among the 10 cancer centers included in the study. Fourth, although the number of cancer patients at each center differed, we included the same number of FCs for each center. Fifth, FCs who completed suicide following suicidal ideation or attempts were not assessed in this study. Finally, due to the small number of FCs who had attempted suicide, we were able to examine only a limited number of factors related to suicide attempts. Despite these limitations, the present study has several important implications for FCs of patients with cancer. To the authors’ knowledge, this is the first published study to investigate the prevalence of suicidal ideation and suicide attempts in FCs of patients with cancer and to identify groups at high risk by identifying factors associated with suicidal ideation and suicide attempts in FCs with anxiety or depression. Although we could not compare the rates of suicidal ideation and attempts among FCs in Korea with those among FCs in other countries because this phenomenon has rarely been examined, one previous study did compare the prevalence of suicidal ideation and attempts in the general population across nine countries. According to the results of that study, the rate of suicidal ideation in the Korean general population was similar to those in the US, West Germany, France, and New Zealand. Additionally, the rates of suicide attempts were consistent across most countries. [34] Therefore, the authors suggest that our findings may be generalizable to countries in which the rates of suicidal ideation and attempts are similar to those in Korea.”

### Conclusions

FCs are essential participants in the delivery of the complex services required for cancer care. Providing support for FCs benefits caregivers, patients, and healthcare teams. FCs have received an increasing amount of attention over the past decade. [35] It is essential to identify and treat caregivers who are experiencing psychological problems, even at a subclinical stage, to relieve stress. Our findings can be used in clinical settings to help clinicians assess the mental health of caregivers, identify those at high risk for suicide, and provide early and timely interventions for FCs. The results of the present study suggest that early detection of anxiety or depression in FCs of patients with cancer will allow interventions, such as providing the opportunity to continue to work during caregiving, that may help manage or prevent suicide. The initiation of workplace policies or programs that help FCs manage their roles as workers and caregivers (e.g., by making sick leave or flexible work hours available) may be necessary to increase the welfare of both patients with cancer and FCs. Additionally, hospital- or community-based interventions that increase the social support for FCs or social status of FCs and increase their perceived QOL may be helpful for improving their mental health. Further studies of the psychological health, personal welfare, and well-being of FCs as well of the behavioral and physical consequences of this role are warranted.
